# Gouty Rheumatism—Lachesis

**Published:** 1884-01

**Authors:** Walter M. James

**Affiliations:** Philadelphia


### GOUTY RHEUMATISM—LACHESIS.

WALTER M. JAMES, M. D., PHILADELPHIA.

A gentleman summoned me to attend him in an attack of gouty rheumatism.

The left foot was much swollen, very sensitive to pressure, and could not sustain the weight of the body. He therefore walked with crutches. No symptoms of any value could at first be found upon which to prescribe except that he had been subjecting himself to the action of quack medicines. *Nux vomica*$^{2c}$ was given, but without effect.

Finally it was observed that the pain began on the *left* side of the foot, and went over to the *right;* that the next day the inflammation extended from the *left foot* to the *right*. Here was a well-known indication for *Lachesis*. This medicine was given with immediate relief. A few days later, the improvement seeming to be fading away, three or four doses of *Lachesis* were given at comparatively short intervals. At once there was a great aggravation of all the symptoms. By the simple expedient of withholding all medicine, the aggravation disappeared and the patient quickly got well.

